# Effects of nest locations on foraging behavior and physiological responses in seabird colony

**DOI:** 10.3389/fphys.2025.1519701

**Published:** 2025-01-31

**Authors:** Yuichi Mizutani, Yusuke Goto, Akiko Shoji, Ken Yoda

**Affiliations:** Graduate School of Environmental Studies, Nagoya University, Nagoya, Japan

**Keywords:** seabird, colony, reproductive cost, antioxidants, pro-oxidant, BAP, d-ROMs, GPS

## Abstract

Breeding success and survival in colonial seabirds are influenced by nest location, physical surroundings, and external disturbances. Nest location may also directly or indirectly affect individual foraging behavior and physiological conditions, shaping reproductive success and survival. Despite these influences, few studies have integrated the analysis of nest location, behavior, and physiological status. In this study, we analyzed 20 black-tailed gulls (*Larus crassirostris*) nesting in the center of a colony within a protected area (Central Group, CG) and five gulls on the periphery outside the protected area, where human disturbance is frequent (Peripheral Group, PG). Using GPS movement trajectories and physiological indicators, we found that although clutch sizes were similar between the CG and PG, the PG exhibited shorter foraging trip durations, maximum distances from the nest, and a lower daily frequency of foraging trips. Antioxidant capacity did not differ between the groups; however, oxidation levels were lower in the PG. These behavioral and physiological differences associated with nest location may partly result from the incubation period influenced by human activity. The PG individuals remained in the peripheral group for at least 2 years (some for over 15 years), with all reproductive attempts failing, suggesting consistently low reproductive success. However, reduced foraging activity and lower oxidative stress levels reflect an energy-saving strategy that may mitigate the costs of repeated breeding failures. These findings suggest a potential life-history trade-off, in which individuals prioritize survival over reproductive success. This highlights how external disturbances and nest location can shape energy allocation strategies within a colony’s peripheral-central distribution.

## 1 Introduction

In colony-forming seabirds, individuals nesting at the center of a colony generally exhibit higher parental survival and reproductive success than those nesting at the periphery, a pattern known as the peripheral-central distribution (Coulson, 1968; Tenaza, 1971; Aebischer and Coulson, 1990; Piro and Schmitz Ornés, 2021). Variations in fitness-related traits across nesting locations may be influenced by internal parental factors, such as age (Aubry et al., 2009), breeding experience (Vergara and Aguirre, 2006), and foraging ability (Spurr, 1975), as well as external factors, including predation risk (Vine, 1971; Brodin, 2001; Brown and Kotler, 2004; Piro and Schmitz Ornés, 2021), physical environmental conditions (Montevecchi, 1978; Rounds et al., 2004; Minias, 2014; Pagenaud et al., 2022), and social interaction levels (Birkhead, 1977; Kokko et al., 2004).

Seabirds may select nesting sites according to these factors, often shifting from subcolonies or peripheral areas to more significant central sites (Serrano and Tella, 2007). As a result, older, more experienced, and reproductively successful individuals are more likely to occupy the central regions, whereas less experienced individuals tend to nest peripherally (Aebischer and Coulson, 1990; Bosch and Sol, 1998; Kim and Monaghan, 2005; Indykiewicz et al., 2019). However, some seabird species exhibit strong nesting site fidelity, returning to the same location in each breeding season (Pearce, 2007; Piro and Schmitz Ornés, 2021), potentially because of their genetic preferences for specific habitats (Rodway and Regehr, 1999).

Although colony position and associated factors can influence reproductive outcomes (but not always; see Ryder and Ryder (1981), Shaw (1985)), the long-term strategies of iteroparous seabirds may buffer against the negative impacts of occasional breeding failures. Temporary breeding failures in peripheral nests may not substantially reduce overall fitness because skipping a breeding attempt allows individuals to conserve resources, including physiological conditions, that can be allocated to future breeding opportunities. To further clarify the peripheral-central nest-site distribution, comparing the physiological loads of parental seabirds, in conjunction with their behavior, between the peripheral and central nests is essential.

One promising approach involves examining oxidative stress, which has become a key indicator of the physiological costs associated with wildlife behavior (Koyama et al., 2021). It arises from an imbalance between pro-oxidants, particularly reactive oxygen species (ROS), and antioxidant defenses (Monaghan et al., 2008). While ROS plays a crucial role in pathogen elimination, their excessive accumulation can damage DNA, proteins, and lipids, leading to fatigue due to impaired mitochondrial function and a shift toward anaerobic metabolism. Conversely, antioxidants mitigate oxidative damage through endogenous enzymes and dietary compounds (Dröge, 2002). Elevated ROS levels are generally associated with physiological strains, whereas antioxidant levels indicate the capacity for recovery and resilience (Ahmadi et al., 2006). In avian studies, female European starlings (*Sturnus vulgaris*) experimentally subjected to increased breeding costs exhibit reduced physiological functions, including increased oxidative stress (Fowler and Williams, 2017). Furthermore, individuals may adjust their antioxidant mechanisms in response to anticipated conditions, potentially balancing endogenous and dietary antioxidant activities based on past environmental experiences and expected intake (Noguera et al., 2015). Oxidative stress has also been observed to exert delayed effects on life-history traits linked to survival (Noguera et al., 2012) and is often positively correlated with reproductive effort (Christe et al., 2012; Fletcher et al., 2013); however, some studies have reported no such correlation (Nussey et al., 2009; Markó et al., 2011).

**FIGURE 1:**
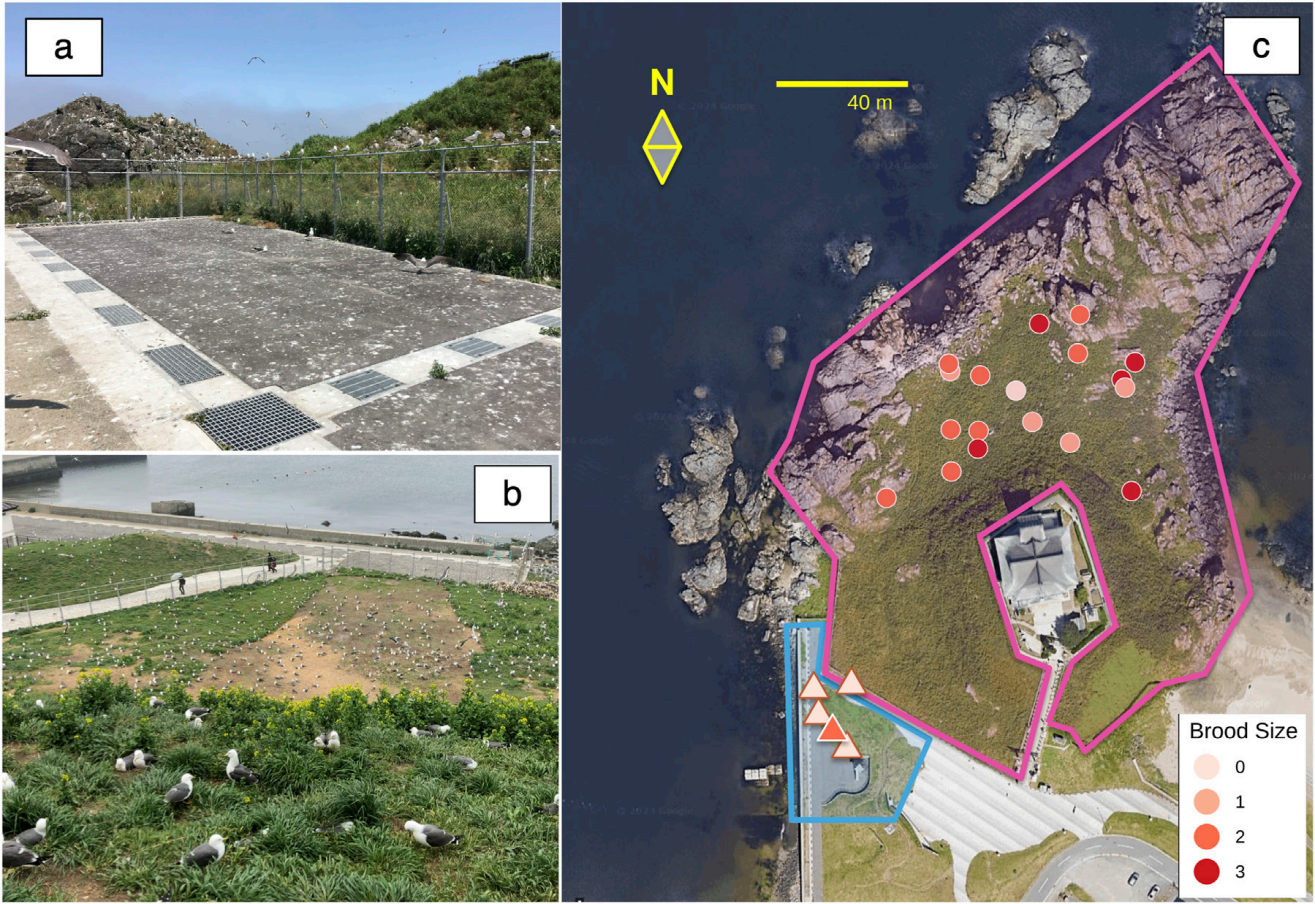
Photographs of Kabushima Island and its surroundings **(A, B)** and an aerial view **(C)** (modified from the “Kabushima Island Black-tailed Gull Breeding Ground” Natural Monument Environmental Survey Report). **(A)** The nesting area is located directly outside the fence and is accessible to visitors. **(B)** View from Kabushima Shrine at the island’s summit (located on the right hill in (a)), showing the protected zone in the foreground and the unprotected area beyond the fence in the background. **(C)** The pink-outlined area marks the protected zone, fenced on the land side and facing the sea, while the blue-outlined area indicates the accessible, unfenced zone used for the peripheral group survey. The nesting locations within the protected area (Central Group, CG) are shown as circles based on GPS-measured coordinates. In contrast, those outside the protected area (Peripheral Group, PG) are shown as triangles, mapped relative to the fence and fence posts. GPS data were unavailable for one CG individual, and one pair was included, resulting in 18 nests displayed for CG.

In this study, we compared the physiological loads and foraging movements of black-tailed gulls (*Larus crassirostris*) incubating on Kabushima Island, where a fenced, sea-surrounded protected center (Central Group, CG) was contrasted with a more exposed periphery accessible to humans and predators (Peripheral Group, PG) (Figure 1). Although defining clear boundaries between the central and peripheral areas is challenging, the fenced structure of this colony allowed us to identify distinct groups consistent with the peripheral-central distribution. We recorded foraging movements using biologging and measured physiological loads based on oxidation levels and antioxidant capacity. Given that sexual differences in foraging behavior (Kazama et al., 2018) and/or antioxidant capacity (Lin et al., 2022) may reflect differences in breeding investment or physical condition, we also compared clutch size and body size, traits often correlated with and indicative of reproductive effort, between the CG and PG. Clutch size, which represents reproductive demand, may also influence oxidative stress because it is related to the heat requirements of seabirds (Biebach, 1984; Moreno et al., 1991; Moreno and Sanz, 1994), including black-tailed gulls (Niizuma et al., 2005). Because black-tailed gulls on Kabushima Island are known to exhibit strong nest site fidelity (Narita and Narita, 2004), we hypothesized that PG individuals may consistently experience lower reproductive success due to interrupted breeding or reduced reproductive investment while simultaneously gaining physiological benefits that enhance self-maintenance and future breeding potential.

## 2 Methods

### 2.1 Ethical note

All fieldwork was conducted by highly skilled personnel who had completed comprehensive training in animal experimentation ethics as required by the Animal Experimental Committee of Nagoya University. All procedures used in this study were approved by the Animal Experimental Committee of Nagoya University (V230001-003). Additionally, protocols for capturing birds on Kabushima Island, a national natural monument, were approved by the Hachinohe City Board of Education (permit number 2023-294) and the Aomori Prefectural Government (permit number 2023-3045) under the Ministry of the Environment approval for equipment installation (2404111).

### 2.2 Field work

This study was conducted at the black-tailed gull breeding site on Kabushima Island, Japan (40°32′20°N, 141°33′26 E) from April to June 2023, coinciding with the breeding season of the species. Intermittent marking surveys have been conducted at the site since 1922. Since 1973, approximately 2,000 chicks have been banded annually with metal rings by the Ministry of the Environment before fledging (Narita and Narita, 2004), allowing for precise age determination and individual identification of many parent birds in the colony. Kabushima Island has been designated a protected natural monument since 1922 and was connected to the mainland in 1943 through land reclamation. The island’s perimeter, shrine precincts, and approach roads are open to visitors. In contrast, the remaining areas, from the midsection to the coast, are fenced and designated as protected zones inaccessible to the public (Figure 1). Approximately 30,000 black-tailed gulls inhabit protected and unprotected areas on and around the island (Biodiversity Center of Japan, 2019). In May 2023, 52 dead individuals were found on and around Kabushima Island. The cause of death was unknown in 50 cases; one case was attributed to predation by a cat, and the other to predation by a bird (FY2024 Kabushima black-tailed gull monitoring report).

The Peripheral Group (PG) consisted of five adult birds marked with metal or numbered plastic rings in 2007 and 2008, nested in unfenced areas or immediately below the fenced region. Individuals were selected based on their rings to ensure a history of consistent nesting in the area. In recent years, no chicks have survived in these areas, and no successful nestlings have been recorded since 2021 (Figure 1C). Observations began during the pre-nesting period, and egg-laying dates, egg measurements, and clutch sizes were recorded upon confirmation of nesting. Parent birds were captured manually during the incubation period. Daily patrols were conducted until all eggs were hatched or 5 days after the expected hatching date. Unhatched eggs were recorded for each nest, and nests with dead chicks or eggshells following the anticipated hatching date were classified as unsuccessful. In 2023, breeding began approximately 2 weeks earlier than in previous years, complicating the determination of exact egg-laying dates for some individuals. For eggs with uncertain laying dates, the incubation periods were estimated based on data from the 2021 and 2022 surveys, in which the laying and hatching dates were confirmed. The incubation period for the first egg was calculated as 28.6 days and standardized to 29 days, whereas that for the second and third eggs was calculated as 26.9 days and standardized to 27 days.

Captured adult birds were weighed, and morphometric measurements were collected. Approximately 700 µL of blood was drawn from the brachial vein, with the area disinfected using a cotton pad soaked in 70% ethanol. Blood was collected using a 25G or 29G syringe (NIPRO, Japan) preloaded with liquid heparin (5,000°units/5 mL; Mochida Pharmaceutical Co., Ltd., Tokyo, Japan) and stored in microtubes for transport to the laboratory. Subsequent experimental procedures were conducted after ensuring hemostasis at the puncture site. Previous studies have indicated that the volume of blood collected has negligible effects on the behavior, reproductive success, and survival of adult black-tailed gulls (Mizutani et al., 2013).

After blood collection, FLEX II Max devices (15.5 g, Druid Technology, China) were attached to the birds using the harness method (Thaxter et al., 2014; Koyama et al., 2024). A Teflon ribbon (TH-25; 6 mm width; BallyRibbonMills, Bally, PA, United States) secured the device on the bird’s back. This method, previously employed in black-tailed gulls at this site, has been shown to have minimal effects on survival, reproduction, and behavior over periods exceeding 1 year (Hibiki Sugiyama *in prep*.).

The FLEX II device, powered by solar energy, transmits data via mobile radio, eliminating the need for recapture. Behavioral data were collected continuously, even after breeding efforts ceased. The device was set to the standard mode with a GNSS positioning interval of one point per hour and a communication interval of 8 hours. For comparison, the same behavioral and physiological surveys were conducted on 20 black-tailed gulls nested in a protected area (CG), with blood samples collected for oxidative stress analysis. For the CG birds, a VHF/GPS logger (PinPoint VHF/GPS with solar panels, 82 mm × 25 mm × 27 mm, 18 g; Lotek Wireless Inc., Canada) was used for biologging using the harness method. GPS data for the CG were collected at 5-minute intervals, allowing for detailed multi-year tracking of individual behavior. Because birds that lose their eggs often abandon their nests, communication devices are attached to PG birds to ensure continuous data collection. For the CG birds, we used individuals equipped with a VHF/GPS logger that our research group had deployed for long-term monitoring over multiple years.

### 2.3 Laboratory procedures: blood processing and oxidative stress assays

The blood samples were transported from the study site to the laboratory in light-protected containers. Within a few hours of collection, samples were centrifuged to separate plasma and hemocyte fractions, which were then stored at −20°C for later oxidative stress assays. DNA was extracted from blood cells obtained after centrifugation using the DNeasy Kit, and sex was determined using a PCR-based method (Fridolfsson and Ellegren, 2000; Mizutani et al., 2016). For oxidative stress measurements, plasma samples were thawed for 1 h before analysis and centrifuged at 14,000 rpm at 4°C for 10 min. A middle plasma layer was used to avoid the contamination of the upper and lower layers. Oxidative stress levels were measured using the Free Carrio Duo system (Diacron International, Grosseto, Italy) with d-ROMs and BAP test reagents, following established protocols to assess oxidative status (d-ROMs in U. CARR) and antioxidant capacity (BAP in µM) (Koyama et al., 2021). To maintain sample integrity, all assays were conducted with samples kept below 10°C. Plasma samples that appeared turbid during measurement were excluded from subsequent analyses and treated as missing values. This affected four individuals in the CG (d-ROM and BAP measurements) and one in the PG (BAP only).

### 2.4 Laboratory procedures: analysis of biologging data

All behavioral and statistical analyses were conducted using R version 4.3.2 (R Development Core Team, 2023). Only high-accuracy biologging data (GNSS accuracy below 7 HDOP or VDOP) were retained for analysis, and missing values were removed. Because two different devices were used, movement data from the FLEX II and VHF/GPS loggers were standardized and resampled at 30-minute intervals using the adehabitatLT package (version 0.3.28; Calenge, 2006). Data points with speeds exceeding 90 km/h, considered errors, were removed using the ddfilter function of the SDLfilter package (version 2.3.3; Shimada, 2023).

As black-tailed gulls engage in central-place foraging during the breeding season, a foraging trip (hereafter “trip”) was defined as any excursion of at least 500 m from the nest that lasted 30 min or more. We defined the distance as the straight-line path between the GPS positioning points recorded by the device and the border between the colony and a nearby location (fishing port) where the birds were expected to stay. The duration, maximum distance from the breeding site, and total distance traveled were calculated as the primary trip parameters for each trip. To ensure consistency within the incubation period, analyses were limited to data collected from May 1st or the device deployment date. This allowed for a more extended period, until the emergence of the first hatchling. As a result, the average analysis periods were 25.4 days (range: 18–31 days) for CG and 21 days (range: 19–23 days) for PG (see supplementary materials).

This study examined whether behavioral and physiological variables differed by nest location and investigated the influence of behavioral parameters (trip duration, maximum distance from the colony, and total trip distance as indicators of foraging effort) and clutch size on oxidative stress markers (d-ROMs and BAP). However, the maximum distance from the colony and the total distance were highly correlated (Pearson correlation coefficients: trip duration and maximum distance, r = −0.214; trip duration and total distance, r = 0.373; maximum distance and total distance, r = 0.747). Because of the wide sampling interval (1 point per 30 min) even after interpolation, the maximum distance from the colony was used as the primary trip distance indicator in subsequent analyses. Bayesian *t*-test was used to evaluate the impact of each variable on oxidative stress levels (d-ROM and BAP). To examine group differences in oxidative stress markers, foraging behaviors during the incubation period (trip duration, maximum reach, total distance, total number of trips during the period, number of days measured, and average number of trips per day based on these figures), and clutch size between the PG and CG, we used Bayes Factor analysis with the BayesFactor package in R (version 0.9.12–4.7; Morey et al., 2024). A Bayes Factor (BF) value greater than one supports the alternative hypothesis, indicating a difference between groups, whereas a value less than one is evidence for the null hypothesis, indicating no difference. In addition, we used BF to confirm the absence of differences between the CG and PG in terms of body mass, and each external measurement was analyzed separately for males and females (see supplementary materials). Moreover, the relationship between clutch size and female body mass was evaluated using a Bayesian linear model, which did not detect a significant relationship between these variables (detailed methods and results are provided in the Supplementary Materials).

## 3 Results

The average clutch size for the five PG gulls (3 females and 2 males) was 2.4 ± 0.54 SD (range: 2–3). No chicks hatched in four of these nests, whereas in one nest, two chicks hatched; however, the first chick disappeared at 5 days post-hatching, and the second chick died at 4 days of age. Although PG parents remained near the nest after these losses, no additional eggs were laid. For the CG, 20 gulls (5 females and 15 males) were caught during the incubation period, with an average clutch size of 2.25 ± 0.79 SD (range: 1–4), an average of 1.86 ± 0.91 SD (range: 0–3) hatched chicks per clutch, and an average of 1.00 ± 0.77 SD (range: 0–3) fledglings (chicks that survived to 30 days of age). In two CG nests, all eggs failed to hatch.

The PG birds were at least 10 years old, including one born in 2006 and another in 2013 (both identified by metal rings). The other three were captured in 2007 and 2008 and identified using plastic rings. The age of the CG birds ranged from 5 to 24.

**FIGURE 2:**
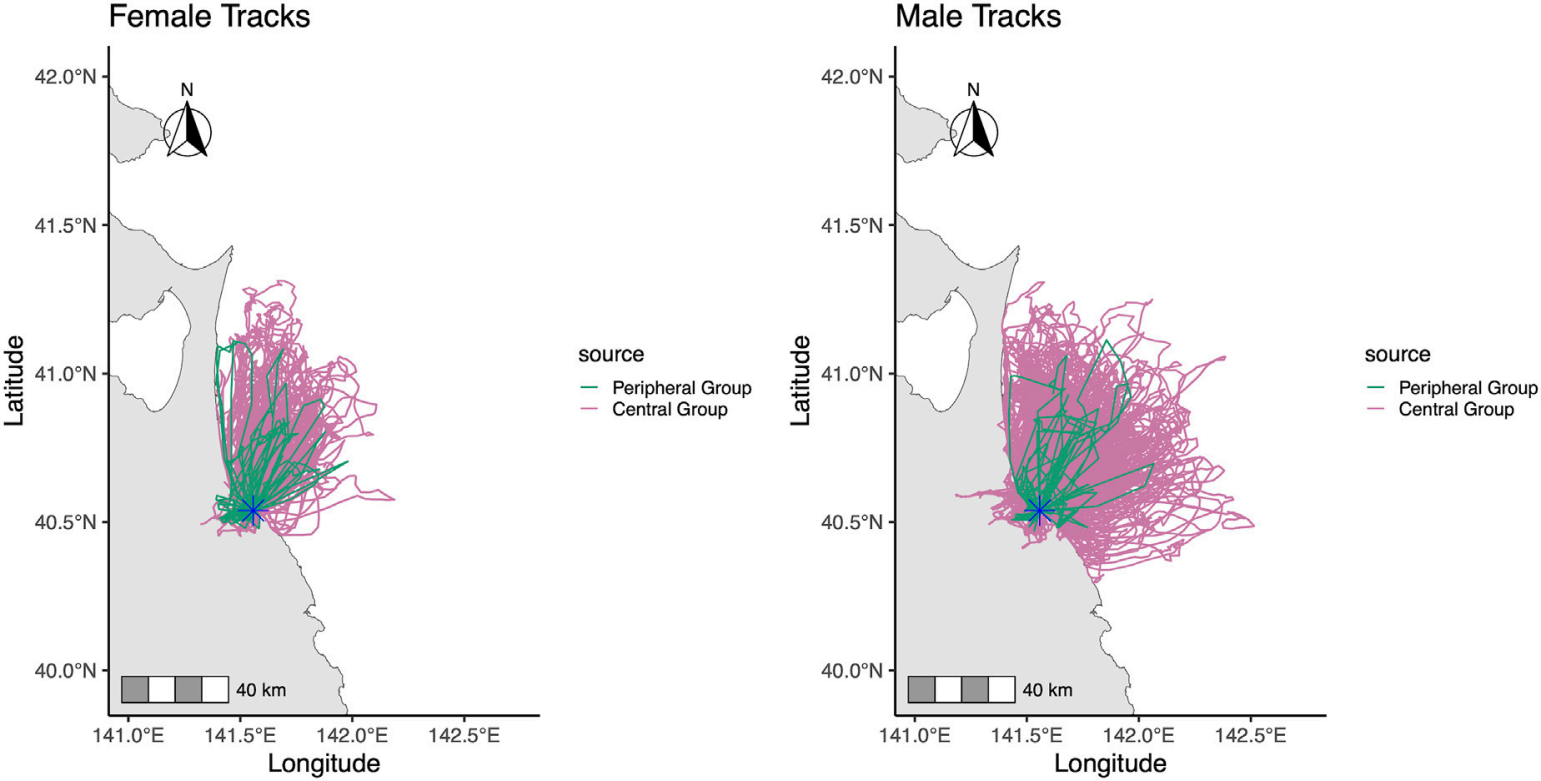
Foraging trip tracks of black-tailed gulls on Kabushima Island during the incubation period. Tracks for the Central Group (red; 20 birds: 5 females, 15 males) and the Peripheral Group (green; 5 birds: 3 females, 2 males) are shown. The asterisk marks the location of Kabushima Island.

**FIGURE 3:**
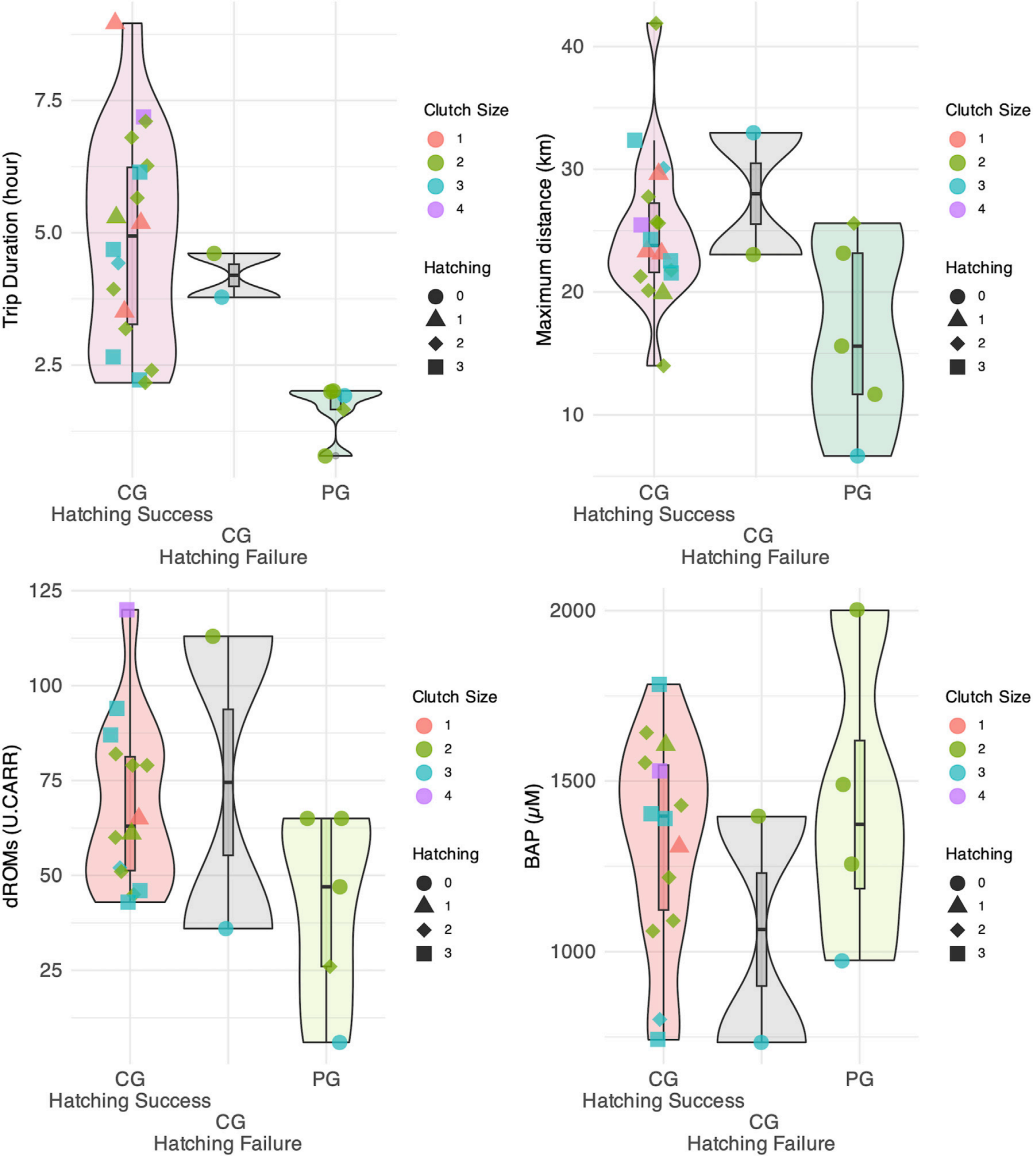
Distribution of trip duration, maximum distance reached from the colony, oxidation levels, and antioxidant capacity for the Peripheral Group (PG) and Central Group (CG). The CG is further divided into groups based on whether egg hatching was successful or not.

The average trip duration and maximum distance traveled were 1.48 ± 3.20 h and 15.72 ± 15.70 km for PG, and 3.26 ± 2.89 h and 24.28 ± 18.89 km for CG (Figures 2, 3). The mean number of daily foraging trips was 3.79 ± 1.24 for CG and 2.75 ± 1.18 for PG, respectively. The mean oxidation levels (d-ROMs) and antioxidant capacity (BAP) were 41.8 ± 25.7 U. CARR and 1,431.0 ± 435.0 µM for PG, and 69.6 ± 25.2 U. CARR and 1,293.0 ± 326.0 µM for CG (Figure 3). In addition, behavioral and oxidative stress measurements of the two CG gulls whose eggs failed to hatch did not deviate from the range observed in the CG group.

The Bayesian *t*-test results showed significant differences in trip duration between the PG and CG, with a Bayes Factor (BF) of 20.97, indicating a strong effect. BF = 4.60 suggested a moderate difference between the groups for the maximum distance traveled. The oxidation level showed weak evidence for a difference with BF = 1.82, whereas the antioxidant capacity (BF = 0.54) showed no difference between the groups. The clutch size also showed no evidence for a difference, with a BF of 0.45. Additionally, the Bayesian *t*-test results for the number of measurement days, the total number of trips, and the average number of trips per day yielded the following BF values: 2.52 for the number of measurement days, providing weak evidence for a difference between the groups, 148.30 for the total number of trips, providing strong evidence for a difference; and 21.90 for the average number of trips per day, also providing strong evidence for a difference.

## 4 Discussion

The PGs could not raise chicks because of several factors, including non-hatching, egg loss, and chick mortality, a pattern similar to that observed in previous years for most parents in peripheral areas. It is unclear whether non-hatching results from unfertilized eggs or inadequate embryonic development. This is because the PG area was easily accessible to tourists and feral cats, frequent disturbances caused parents to leave their nests. These disturbances are likely to reduce hatching success, either through direct nest attacks or lowered nest temperatures linked to shortened incubation periods (Di Giovanni et al., 2022; Clemencin et al., 2024), as well as increased stress resulting from proximity to disturbances (Ellenberg et al., 2007).

Regardless of hatching success, the antioxidant capacity was similar between the PG and CG, indicating that the intra-species variation in antioxidant capacity was slight. This finding suggests that black-tailed gulls maintain comparable levels of non-enzymatic endogenous antioxidants (e.g., vitamins A and E, uric acid; Sharma et al., 2018) and exogenous antioxidants (e.g., coenzyme Q10; Zozina et al., 2018) and carotenoids (Milani et al., 2017). In contrast, differences in foraging movements and oxidative stress levels suggest that the primary defense against oxidative stress during the breeding season likely depends on endogenous enzymes such as glutathione peroxidase. Similar to other bird species (McWilliams et al., 2021), glutathione peroxidase may serve as the first line of defense against ROS. Consequently, if the observed levels of foraging and breeding efforts remain within the capacity of endogenous enzymatic systems, the immediate need for additional exogenous antioxidants may be minimal.

Compared to the CG, the PG exhibited significantly shorter trip durations, a smaller maximum distance reached (strongly correlated with the total distance), and a lower frequency of foraging trips. Black-tailed gulls on Kabushima Island utilize a variety of feeding grounds and consume a broad diet beyond fish and shellfish (Yoda et al., 2012; Mizutani et al., 2021). Their regurgitated food items include freshwater fish and insects (Narita and Narita, 2004). Although the exact types and quantities of food consumed in this study remain unknown, the foraging range of PG overlapped substantially with that of CG, and no differences in antioxidant levels were observed between the groups. This suggests that both the PG and CG consumed similar types and amounts of food. However, the observed differences in foraging behavior likely influenced energy expenditure, leading to slight differences in ROS production and oxidation levels.

Although antioxidant levels were similar between individuals, PG exhibited lower oxidation levels, possibly due to nearby, shorter foraging trips and a lower daily foraging frequency. Gull species spend much of their flying time flapping their wings, a highly energy-intensive activity (Bishop, 2005; Mizrahy-Rewald et al., 2022) that likely increases energy consumption and ROS production. Additionally, the incubation period imposes significant physiological costs on the parent birds. For example, in common eiders (*Somateria mollissima*), high incubation demands lead to weight loss and decreased immune function due to reduced food intake, resulting in decreased fertility in the following year and long-term fitness costs (Hanssen et al., 2005). In contrast, the failed hatching observed in PG suggests inadequate incubation, which would have led to lower incubation costs. Although breeding costs are generally buffered during the non-breeding season (Senner et al., 2014; Briedis et al., 2018; Gow et al., 2019), consistently reduced breeding investment may minimize negative carryover effects in subsequent seasons.

PG individuals experienced consistently low reproductive success over multiple years; however, it is intriguing that they do not change their nesting locations. They may lack the competitive ability to secure favorable nest sites. Instead, the low oxidative stress associated with less demanding foraging trips may offset the high reproductive costs borne by CG individuals, reflecting an underlying life-history trade-off. Our results indicate that a peripheral-central distribution generates heterogeneity in reproductive success within a colony. However, the peripheral groups may gain fitness benefits by occasionally foregoing reproduction. Future research should investigate behavioral differences during the incubation, chick-rearing, and non-breeding periods, as well as their long-term physiological costs. Such investigations would enable a more comprehensive evaluation of rearing costs within the broader framework of life-history trade-offs. From a conservation perspective, completely fencing off all black-tailed gull nesting areas to protect PG groups from predators and human disturbances may be impractical. Instead, phased measures, such as deterrents against easily accessible small terrestrial mammals and visitor restrictions, combined with countermeasures such as fencing, which are considered critical to success, are expected to improve the hatching success rate of PGs.

One limitation of this study is the absence of continuously recorded physiological data. Blood, a vital biomarker source, is widely used in diagnostics and research (Toner and Irimia, 2005), underscoring the significant demand for real-time blood sampling loggers. Wearable automatic blood-sampling devices designed to minimize pain and distress are being developed for use in captivity settings (Li et al., 2009; Li et al., 2015; Hopper, 2016). While the long-term collection of blood from wild flying animals remains challenging, advances in non-invasive techniques, such as continuous glucose monitoring via interstitial fluid (Mathew et al., 2024), offer promising alternatives. Deploying such technologies in seabirds could revolutionize biologging, enabling researchers to explore how species balance reproduction and survival, respond to environmental disturbances, and allocate energy across life-history stages.

## Data Availability

The raw data supporting the conclusions of this article will be made available by the authors, without undue reservation.
