# Supplementary material for: Effects of nest locations on foraging behavior and physiological responses in seabird colony
Source: Front Physiol. 2025 Jan 31;16:1519701. doi: 10.3389/fphys.2025.1519701 (PMC11825827; doi:10.3389/fphys.2025.1519701)
Supplement: Supplementary file 1 [file Table1.docx]

**Supplementary materials**

"Effects of nest locations on foraging behavior and physiological responses in seabird colony"

**Statical analysis**

1. We compared the differences between the central group (CG) and peripheral group (PG) in body mass and external measurements using the Bayes Factor package in R (version 0.9.12-4.7; (Morey et al. 2024). The comparison was conducted separately for males and females because there are differences in the body size of black-tailed gulls. We compared the body mass, bill depth, head length, tarsus length, and natural wing length between male and female CG and PG birds. The null hypothesis assumed no differences between the CG and PG birds in terms of body mass and external measurements, whereas the alternative hypothesis posited the presence of such differences. Bayes Factors (BF) were calculated to assess the relative support for these hypotheses, with BF values <1 indicating evidence favoring the null hypothesis and values >1 indicating evidence favoring the alternative hypothesis.

2. We used the Bayesian linear model in the Brms package (ver. 2.32.6 (Guo et al. 2024)) to evaluate the relationship between female body mass and the number of eggs per clutch. The BM was modeled using the Student’s t-distribution to address potential outliers. The model treated C.S. as a categorical variable, and sampling was performed with 2000 iterations across four chains using cmdstanr (ver. 0.8.1,(Gabry et al. 2024)) backend." Convergence was assessed using the Gelman-Rubin diagnostic (𝑅^ < 1.1). The model estimates provided the mean and 95% credible intervals for the body mass of each group.

**Result**

1. The results of evaluating the hypothesis that “there is no difference between the CG and PG groups” for each sex were as follows. For males, the BF for each index between the CG and PG was as follows: The BF for body weight was 2.184, suggesting a weak difference between the CG and PG groups. The BF for the bill depth was 3.118, suggesting a moderate difference between the groups. The BFs for head length, tarsus length, and natural wing length were 0.635, 0.621, and 0.612, respectively, with little evidence supporting the difference between the groups. For females, the BF for each index between the CG and PG were as follows: The BF for body weight, bill depth was 0.534, head length was 0.703, tarsus length was 0.533, and natural wing length were 0.532, 0.534, 0.703, 0.533, and 0.690, respectively. As the BF for all these was less than 1, there was little evidence to support the hypothesis of a difference between the CG and PG groups.

2. The results of a Bayesian linear model evaluating the relationship between clutch size and female body mass indicated that the average body mass of the population with a clutch size of 1 was 564.79 g (95% credible interval: [543.41, 586.11]). g(-9.80, 9.51) and 0.01 g(-9.53, 9.62) for populations with 2 or 3 eggs per clutch, respectively. However, evidence supporting these findings is insufficient.

**Reference list**

Gabry, J., R. Češnovar, A. Johnson, and S. Bronder. 2024. *Cmdstanr: R Interface to “CmdStan”. R Package Version 0.8.1*. https://mc-stan.org/cmdstanr/.

Guo, Jiqiang, Jonah Gabry, Ben Goodrich, Andrew Johnson, Sebastian Weber, Hamada S. Badr, Daniel Lee, et al. 2024. *R Interface to Stan [R Package Rstan Version 2.32.6]*. Comprehensive R Archive Network (CRAN). https://CRAN.R-project.org/package=rstan.

Morey, Richard D., Jeffrey N. Rouder, Tahira Jamil, Simon Urbanek, Karl Forner, and Alexander Ly. 2024. *Computation of Bayes Factors for Common Designs [R Package BayesFactor Version 0.9.12-4.7]*. Comprehensive R Archive Network (CRAN). https://CRAN.R-project.org/package=BayesFactor.

Narita Kiichi, and Narita Akira. 2004. *Observation reports of black-tailed gull: Kabushima-island Hachinohe city (in Japanese)*. Hachinohe: Kimura-sShoten.

**The results of biologging**
